# Protein nanomechanics in biological context

**DOI:** 10.1007/s12551-021-00822-9

**Published:** 2021-08-07

**Authors:** Jorge Alegre-Cebollada

**Affiliations:** Centro Nacional de Investigaciones Cardiovasculares (CNIC), 28029 Madrid, Spain

**Keywords:** Single-molecule, Titin, Atomic force microscopy, Magnetic tweezers, Optical tweezers, Mechanobiology

## Abstract

How proteins respond to pulling forces, or protein nanomechanics, is a key contributor to the form and function of biological systems. Indeed, the conventional view that proteins are able to diffuse in solution does not apply to the many polypeptides that are anchored to rigid supramolecular structures. These tethered proteins typically have important mechanical roles that enable cells to generate, sense, and transduce mechanical forces. To fully comprehend the interplay between mechanical forces and biology, we must understand how protein nanomechanics emerge in living matter. This endeavor is definitely challenging and only recently has it started to appear tractable. Here, I introduce the main in vitro single-molecule biophysics methods that have been instrumental to investigate protein nanomechanics over the last 2 decades. Then, I present the contemporary view on how mechanical force shapes the free energy of tethered proteins, as well as the effect of biological factors such as post-translational modifications and mutations. To illustrate the contribution of protein nanomechanics to biological function, I review current knowledge on the mechanobiology of selected muscle and cell adhesion proteins including titin, talin, and bacterial pilins. Finally, I discuss emerging methods to modulate protein nanomechanics in living matter, for instance by inducing specific mechanical loss-of-function (mLOF). By interrogating biological systems in a causative manner, these new tools can contribute to further place protein nanomechanics in a biological context.

## Mechanical forces in biology: the role of protein nanomechanics

Biological systems generate and respond to mechanical forces, determining cell and tissue behavior in health and disease (Guck 2019; Hannezo and Heisenberg 2019; Matamoro-Vidal and Levayer 2019; Roca-Cusachs et al. 2017; Saucerman et al. 2019; Vining and Mooney 2017; Zhu et al. 2019). Classical examples include muscle atrophy induced by long-duration spaceflights (Fitts et al. 2010), brain damage caused by concussion events (Hirad et al. 2019), and cardiac hypertrophy due to elevated blood pressure (Drazner 2011). The interplay between mechanical forces and biology involves processes of active force generation by cells but also dedicated mechanisms that sense (mechanosensing) and translate (mechanotransduction) mechanical forces into the language of the cell, which is written in biochemical and metabolic words (Saucerman et al. 2019). The field of mechanobiology, which is concerned with the study of mechanical forces in biology at the molecular, cellular, and organismal scales, has already led to several paradigm shifts. These include the observation that stem cell differentiation is determined by the stiffness of the extracellular matrix (ECM) (Engler et al. 2006), that mechanical forces are fundamental for development (Mammoto et al. 2013; Petridou et al. 2017), that changes in cell and tissue mechanics are important for the onset and evolution of diseases like cancer (Broders-Bondon et al. 2018), that gene expression depends on the mechanics of the nucleus (Shin et al. 2018; Tajik et al. 2016), and that during some bacterial infections, the fight between host and pathogen is mainly mechanical (Persat et al. 2015). These new insights have been enabled by several technological developments, including production of cell-culture-compatible hydrogels with tunable mechanical properties (Caliari and Burdick 2016), force-sensing methods at the cellular (Cost et al. 2019; Prevedel et al. 2019) and molecular (Neuman and Nagy 2008) levels, tools to probe cell mechanics (Roca-Cusachs et al. 2017), and nano- and microfabrication of cell substrates with controlled geometries and their integration into microfluidics platforms for 2D and 3D cell culture (Castiaux et al. 2019; Ermis et al. 2018).

**Figure 1.**
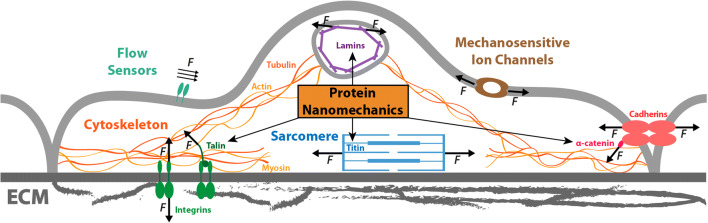
The role of protein nanomechanics in cell mechanosensing, mechanotransduction, and force generation. Cells exploit different mechano-active signaling pathways to produce, sense, and react to mechanical forces. Specialized membrane proteins can sense flow and membrane tension resulting in biochemical signals and/or changes in membrane permeability. Force generation relies on cytoskeletal machines. Among them, sarcomeres present in striated myocytes are a paradigmatic example. Protein nanomechanics is a main contributor to several of these pathways, as exemplified by the role played by titin in force production by sarcomeres, the contribution of lamin to the mechanical integrity of the nucleus and the genome, or the mechanosensing function of integrins and talin in cell/ECM interaction, and that of cadherins/α-catenin in cell-cell junctions

The evolving view is that the landscape of force sensing and force generation mechanisms by cells is broad, highlighting the fact that cells have to ensure correct processing and integration of different mechanical signals for optimal fitness, proliferation, differentiation, and migration (De Pascalis and Etienne-Manneville 2017; Echarri et al. 2019; Hannezo and Heisenberg 2019; Matamoro-Vidal and Levayer 2019; Roca-Cusachs et al. 2017; Saucerman et al. 2019; Yim and Sheetz 2012). Several molecular mechanisms contribute to mechanosensing and mechanotransduction, including membrane tension sensing by mechanosensitive ion channels, flow sensing by extracellular mechanosensors, modulation of the actomyosin cytoskeleton, and force sensing by load-bearing, tethered proteins (del Rio et al. 2009; Douguet and Honoré 2019; Echarri et al. 2019; Fu et al. 2017; Murrell et al. 2015; Orr et al. 2006; Puchner et al. 2008; Schönfelder et al. 2018; Valle-Orero et al. 2017a) (Figure 1). This review focuses on the behavior of proteins under force, or protein nanomechanics, which is relevant not only to understanding protein-based mechanosensing but also organelle integrity, cell adhesion, and muscle function (Figure 1). For over 2 decades, and using different single-molecule approaches, the protein nanomechanics field has gathered extensive information on how force affects the conformational dynamics of proteins. This knowledge has generated hypotheses about how protein nanomechanics influence biology and vice versa; however, these hypotheses have been difficult to test experimentally in a direct manner due to the lack of appropriate tools. In the next sections, I introduce experimental approaches to study protein nanomechanics and summarize fundamental concepts that are now well established in the field, including how different factors influence the free energy landscape of a protein under force. Finally, I review emerging developments to examine the role of protein nanomechanics in living systems. Many laboratories have contributed to the remarkable expansion of the protein nanomechanics field. I have tried to cite the most relevant advances, but I must apologize for any unintended omission.

## Single-molecule force spectroscopy methods to study protein nanomechanics

Not every protein in its biological context is free to diffuse. On the contrary, a fair fraction of them are naturally tethered to cytoskeletal structures, organelles, and/or the ECM and, consequently, subject to mechanical force. The physics of a protein under force has been unraveled thanks to single-molecule force spectroscopy methods (Mora et al. 2020; Schönfelder et al. 2018). Inspired by theoretical predictions on how an end-to-end mechanical force could change the conformation of a protein (Erickson 1994), three reports used single-molecule atomic force microcopy (AFM) and optical tweezers (OT) methods to demonstrate mechanical unfolding and extension transitions in domains belonging to the giant sarcomeric protein titin (Kellermayer et al. 1997; Rief et al. 1997; Tskhovrebova et al. 1997). For the ensuing 2 decades, AFM became the gold standard method to characterize protein nanomechanics, thanks to its relative simplicity and high throughput (Popa et al. 2013b; Yang et al. 2020b). OT, although more experimentally challenging, offers higher force sensitivity and temporal resolution (Moffitt et al. 2008; Neuman and Nagy 2008). More recently, magnetic tweezers (MT) have reached the same level of sensitivity as OT but with remarkable instrumental stability that enables week-long studies on the same protein (Popa et al. 2016). Another advantage of MT is that data acquisition can be parallelized (Lof et al. 2019). To the best of my knowledge, other force spectroscopy methods with parallelization capabilities, including centrifugal force spectroscopy (Yang et al. 2016) and acoustic force spectroscopy (Ozcelik et al. 2018; Sitters et al. 2015), have yet to be applied to the study of protein nanomechanics.

**Figure 2.**
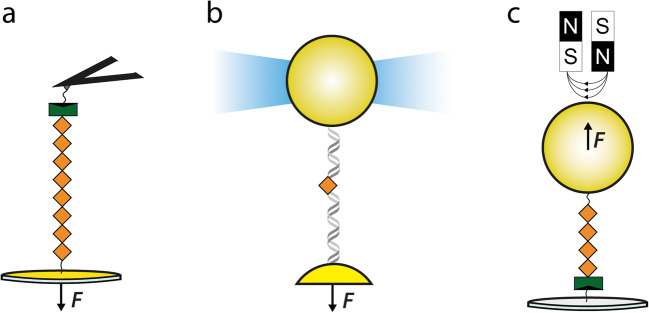
Single-molecule force spectroscopy methods to probe protein nanomechanics. **a** Atomic force microscopy (AFM). An octameric repeat of the protein of interest (in orange) is tethered between the sharp tip at the end of a flexible cantilever (in black) and a cover slip placed on top of a piezoelectric actuator. Retraction of the actuator generates a pulling force, which is sensed from the bending of the cantilever. A covalent anchoring to the cantilever (e.g., HaloTag-based) is shown in green and black. Attachment to the cover slip can be established by different methods including Cys-Au bonds (represented here) or through polyHistidine/Ni-NTA interactions. **b** Optical tweezers (OT). The protein of interest is anchored to two polystyrene beads through DNA handles taking advantage of non-covalent interactions such as biotin/streptavidin (not shown). In OT, at least one bead is trapped by highly focused laser light (blue). Retraction of the other bead, which can be attached to a micropipette or alternatively also trapped by light, generates a pulling force. Force is measured from the position of the bead in the laser trap. **c** Magnetic tweezers (MT). A tetrameric repeat of the protein of interest is tethered between a glass surface (covalent anchoring is represented here) and to a paramagnetic bead. Attachment to the bead can be achieved using different covalent and non-covalent chemistries (not shown). Force is produced by a magnetic field generated in the proximity of the sample chamber.

The experimental design in AFM, OT, and MT is similar (Figure 2). In the three techniques, a purified protein is tethered between a fixed surface and a mobile element, whose displacement results in the application of mechanical force to the anchored protein. In AFM, the protein of interest is tethered between the tip of an AFM cantilever and a surface that can be retracted with sub-nanometer precision, thanks to piezoelectric actuators (Figure 2a). Piezo-driven retraction results in the extension of the tethered protein, and the concomitant bending of the cantilever, which upon calibration, can be used to calculate the force experienced by the protein (Slattery et al. 2014). In AFM, the use of molecular fingerprints, typically based on the repetitive unfolding of serially linked protein domains, ensures identification of successful single-molecule events (Li et al. 2000b; Li et al. 2005). In addition, feedback systems enable force-clamp AFM measurements by adjusting the extension of the piezoelectric actuator to achieve the desired force set point (Schlierf et al. 2004). In pulling experiments by OT, the protein of interest needs to be derivatized to include DNA handles, which are then attached to micrometer-sized polystyrene beads (Cecconi et al. 2005). In a typical OT experiment, one of the beads is trapped by a highly focused laser beam, while the other one is suctioned by a micropipette (or, alternatively, also trapped by laser light) (Figure 2b) (Moffitt et al. 2008). Displacement of the laser trap relative to the micropipette strains the DNA/protein adduct. Force is determined from the position of the trapped beads, and as in AFM, there is possibility to achieve constant force measurements (Moffitt et al. 2008). Time resolution in OT can be as low as a few μs (Neupane et al. 2016). In MT, the protein of interest is tethered to a glass surface and a paramagnetic micrometer-sized polystyrene bead (Liu et al. 2009). Force is applied by approaching a magnet to the sample, which results in tether extension (Figure 2c). The relative position of the bead with respect to reference beads glued to the surface is determined from the diffraction pattern of the beads under a light microscope (Popa et al. 2016). This image analysis is time-consuming and results in limited bandwidth. An advantage of MT over OT and AFM is that there is no requirement for feedback systems to measure at constant force.

The field of force spectroscopy has greatly benefited from computational approaches, in particular Steered Molecular Dynamics (SMD) simulations (Do et al. 2018; Marszalek et al. 1999). By simulating the effect of pulling forces on proteins, SMD methods have the ability to identify unfolding pathways and intermediate states (mechanical clamps) (Marszalek et al. 1999). Importantly, SMD can be used to probe pulling geometries that are not accessible in experiments (Echarri et al. 2019). By defining force fields and protein models to different degrees of detail, simulations can reach several levels of precision and speed. The partnership between SMD and experimental force spectroscopy has become commonplace to investigate protein nanomechanics (Echarri et al. 2019; Milles et al. 2018a; Suay-Corredera et al. 2021b). However, it is important to realize that computational constraints in SMD result in simulated timescales that can differ from those probed experimentally by several orders of magnitude.

Force spectroscopy methods are in constant evolution. For instance, force sensitivity and time resolution in AFM can be greatly improved by the use of carefully designed, very soft cantilevers (Edwards et al. 2017; Yu et al. 2017). High-speed AFM setups can now study protein mechanical unfolding at mm/s speed, which is in the range probed in SMD simulations (Rico et al. 2013). A challenge in force spectroscopy by AFM results from inaccuracies in cantilever calibration (Wagner et al. 2011). However, it has been demonstrated that the accuracy of relative AFM measurements can be increased by concomitant measurements, which can be achieved by multiplexing (Otten et al. 2014) or by orthogonal fingerprinting strategies (Pimenta-Lopes et al. 2019). Recent instrumental development in OT enables simultaneous measurement of fluorescence during mechanical unfolding/folding transitions (Ganim and Rief 2017). Of note, recent ultra-resolution optical tweezer–based measurement of germanium nanospheres has indicated the possibility of examining protein nanomechanics with unprecedented detail (Sudhakar et al. 2021). Regarding MT, optimization can push bandwidth over 10 kHz (Tapia-Rojo et al. 2019). Indeed, in combination with magnetic tape heads to trigger fast force modulation, MT can capture short-lived states in protein folding (Tapia-Rojo et al. 2019) and the response of protein mechanosensors to well-defined force perturbations (Tapia-Rojo et al. 2020b).

Implementation of efficient tethering strategies has been key to the success of force spectroscopy, and it is still a very active area of research (reviewed in Yang et al. 2020b). Different from MT and OT, AFM measurements can be carried out by using non-specific tethering strategies (Echelman et al. 2016; Rief et al. 1997). However, the development of specific tethering methods has dramatically improved both the yield and quality of data acquisition in AFM (Yang et al. 2020b). Non-covalent tethering methods are widespread and include systems based on biotin-avidin/streptavidin binding (Cecconi et al. 2005; Rivas-Pardo et al. 2016), polyHistidine/Ni-NTA interaction (Alsteens et al. 2013), or on specific recognition by antibodies (Rivas-Pardo et al. 2016). A disadvantage of non-covalent tethering strategies is their limited resistance to pulling forces, which poses a problem when studying mechanically stable proteins (Echelman et al. 2016). Notable exceptions include the high stability of cohesin-dockerin and SdvG-Fgβ interactions (Milles et al. 2018a; Schoeler et al. 2014). Alternatively, a handful of specific covalent tethering methods leading to well-defined pulling geometries have been demonstrated and used in AFM and MT experiments. These methods exploit thiol-gold, thiol-maleimide, HaloTag (Popa et al. 2013a; Taniguchi and Kawakami 2010), sortase, ybbR-CoA (Durner et al. 2017), OaAEP1 (Deng et al. 2019), SNAP tag (Kufer et al. 2005), non-canonical amino acid (Yang et al. 2020a), and isopeptide bond (Alonso-Caballero et al. 2021; Zakeri et al. 2012) chemistries. Given the complementary information that can be obtained by AFM, OT, and MT, looking to the future, it will be interesting to develop specific covalent tethering strategies that can probe the same protein preparation with different force spectroscopy techniques.

## The free energy of a protein under force

**Figure 3.**
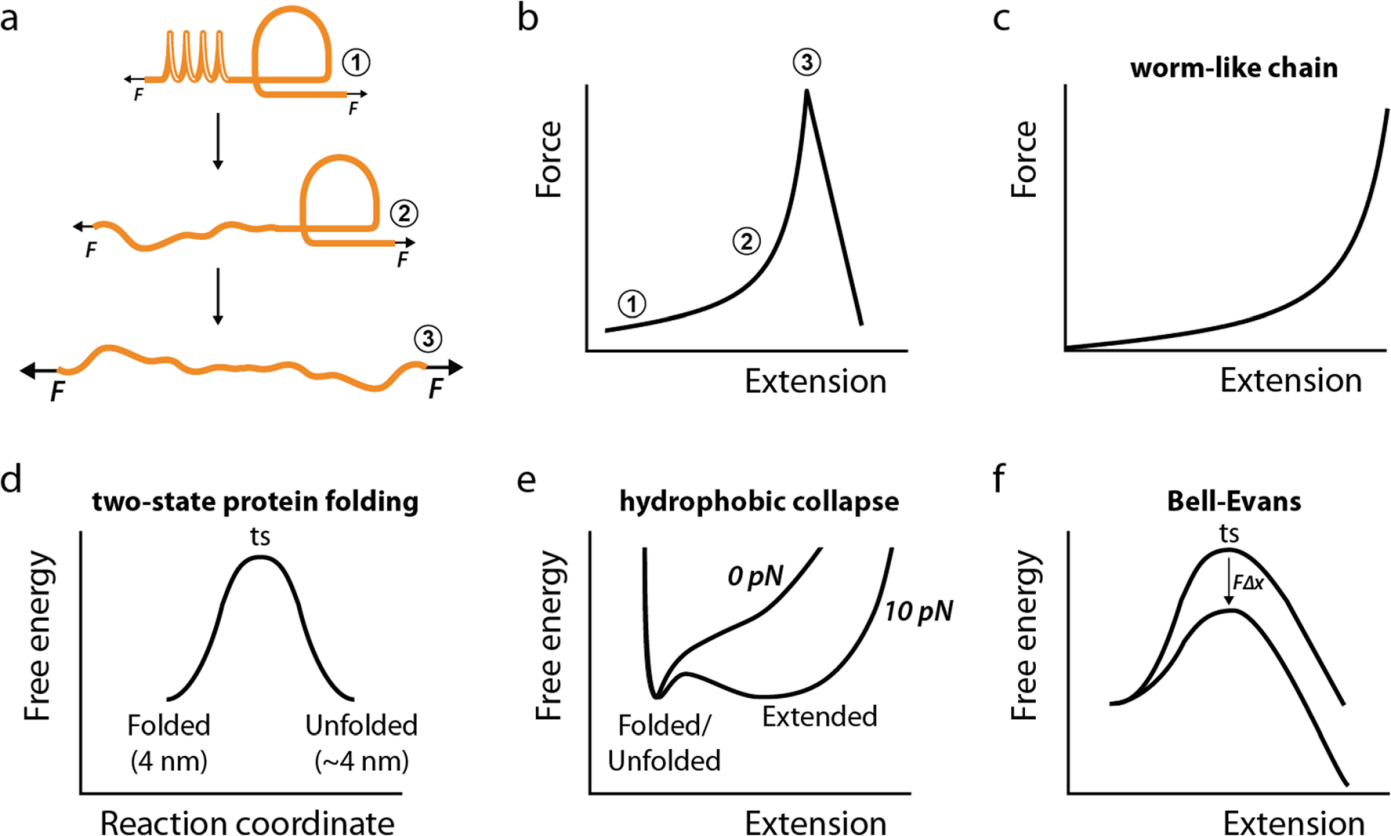
Protein nanomechanics 101. **a** Simplified representation of a protein under mechanical force, which depicts a random coil region as a spring and a folded domain as a loop. The random coil region adapts its length to force in an elastic manner (states 1 through 3), while the folded domain only unfolds at high forces (states 2→3). **b** States 1 through 3 in panel (**a**) are identified in a simulated single-molecule force-extension plot. **c** Graphical representation of force-polymer length relationship according to the worm-like chain model. **d** Simple two-state free energy diagram underlying protein unfolding in the bulk. Please note that the contour length of the folded and unfolded states is very similar. The position of the transition state (ts) is indicated. **e** Simple free energy diagrams of a protein at 0 and 10 pN pulling force considering extension as the reaction coordinate. Extended states can only be observed at forces that overcome the tendency of polypeptides to undergo hydrophobic collapse (i.e., there is no free energy minimum corresponding to an extended state at 0 pN). Since the folded/unfolded states have very similar contour lengths, they cannot be observed directly in single-molecule pulling experiments. **f** Graphical representation of the Bell-Evans model, which considers that the height of the energy barrier decreases by *F*∆*x* when force is applied in the direction of molecular extension. The position of the transition state (ts) is indicated.

Force spectroscopy methods have shown that tethered proteins are more than rigid scaffolds. In simple terms, the response of proteins to a pulling force can be split into entropic and enthalpic contributions (Figure 3a, b) (Li et al. 2002). In the former, random coil polypeptide regions and serially linked protein domains behave as simple springs that adapt their extension (*x*) to the pulling force (*F*) in an elastic manner. Indeed, the elastic behavior of random coil polypeptides follows predictions by polymer physics models such as the worm-like chain (Bustamante et al. 1994) (Figure 3c), which is given by:

1 $$ F(x)=\frac{k_BT}{L_p}\left[\frac{1}{4}{\left(1-\frac{x}{L_c}\right)}^{-2}-\frac{1}{4}+\frac{x}{L_c}\right] $$

In Equation 1, *k*_*B*_ is the Boltzmann constant, *T* is the absolute temperature, and *L*_*p*_ and *L*_*c*_ are the persistence and contour lengths of the polymer under strain. Regarding enthalpic contributions, folded protein domains experience reversible unfolding transitions that are highly force dependent (Schlierf et al. 2004) (Figure 3a), and that can lead to modulation of downstream signaling via differential exposure of binding sites (del Rio et al. 2009).

Several theoretical developments have been put forward to reconstruct underlying force-dependent free energy landscapes from force spectroscopy data (Mora et al. 2020; Valle-Orero et al. 2017a). In this regard, two main considerations need to be taken into account. First, an equilibrium exists between folded and unfolded, but still compact, states of proteins that applies also to tethered proteins (Garcia-Manyes et al. 2009a; Tapia-Rojo et al. 2019). These transitions between states with very similar end-to-end lengths may not be observed from force spectroscopy recordings directly (Figure 3d, e) (Rivas-Pardo et al. 2016). In addition, extended states of polypeptides can only be reached upon a threshold pulling force that overcomes their tendency to collapse by hydrophobic interactions (Figure 3e) (Berkovich et al. 2010a; Berkovich et al. 2010b; Walther et al. 2007).

For many proteins, the rate of mechanical unfolding measured by AFM and MT has been shown to be exponentially dependent on force, in agreement with the simple Bell-Evans model (Bell 1978; Liu et al. 2009; Popa et al. 2013a; Schlierf et al. 2004). This model considers that mechanical force tilts energy barriers according to the work it develops along the reaction coordinate, resulting in an exponential dependency of transition rates (*r*) (Figure 3f):

2 $$ r={r}_o\cdotp {e}^{F\cdotp \Delta  x/{k}_B\cdotp T} $$

where *r*_*o*_ is the transition rate in the absence of force and ∆*x* is the distance to the transition state. A modification of the Bell-Evans model considers that the position of the transition state changes with force, which can explain deviations from simple exponential behavior at extreme forces (Dudko et al. 2008). In this regard, the I27 domain of titin has been shown to mechanically unfold at much higher rates than expected when pulled at forces <100 pN using MT (Yuan et al. 2017). It is important to stress that when pulling experiments are conducted at forces higher than a few pN, collapsed states rapidly transition to extended states. Hence, measured transition rates are mostly dependent on the height of the energy barrier between native and unfolded, but still collapsed, states. In this regard, more complex free energy models account for coordinate reactions that are not directly observable in experiments (Dudko et al. 2008) or for the heterogeneity of transition paths due to static and dynamic disorder (Costescu et al. 2017; Kuo et al. 2010). Theoretical developments have also been extended to serially linked arrays of domains, a configuration found in many proteins with mechanical roles (Berkovich et al. 2018; Chetrit et al. 2020; Valle-Orero et al. 2015).

## Modulation of protein nanomechanics

Multiple factors influence protein nanomechanics. Some of these modulators are reversible and appear to be exploited by cells to achieve functional adaptation. In this section, I summarize the best-known determinants of protein nanomechanics.

**Figure 4.**
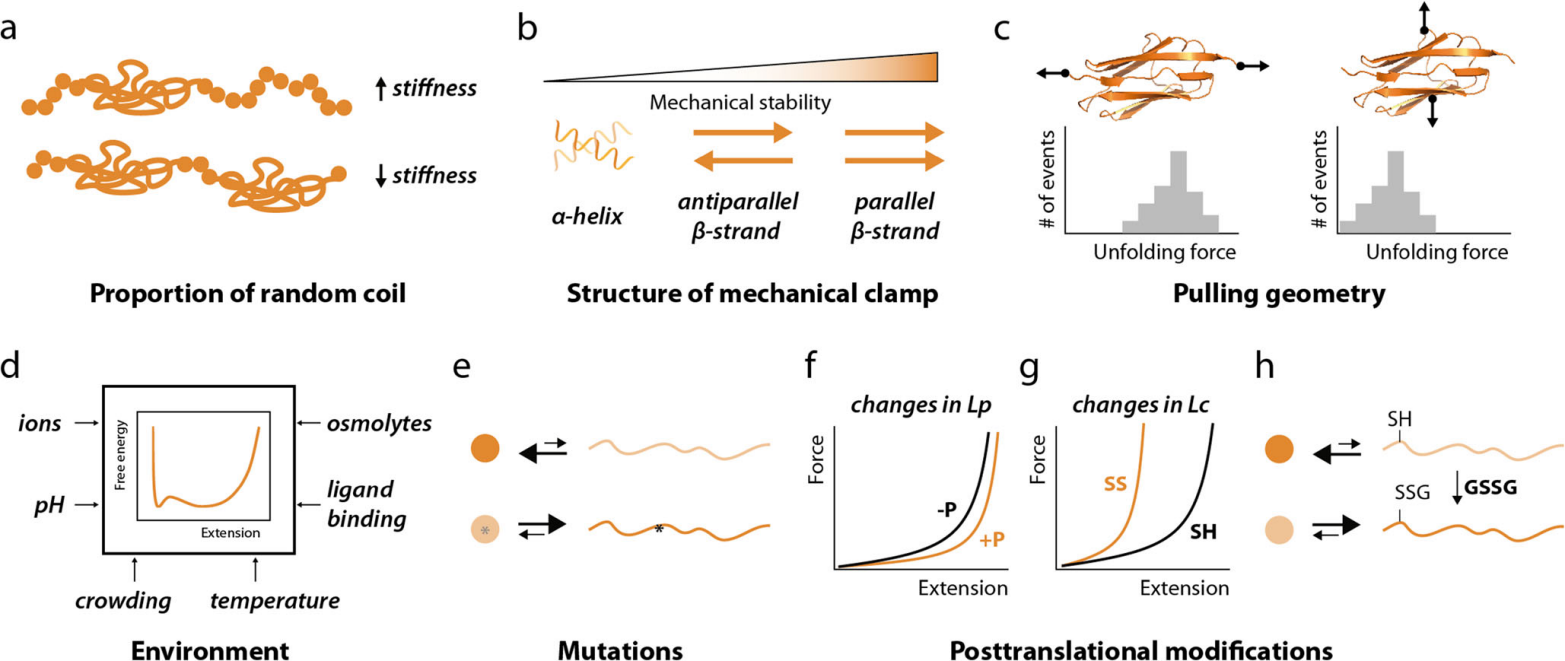
Modulators of protein nanomechanics. **a** Proportion of random coil set the stiffness of proteins under mechanical force. **b** The structure of mechanical clamps determines protein mechanical stability. **c** Illustrative example histograms of unfolding forces of the same protein pulled from two different geometries. **d** The environmental factors in this panel have all been shown to affect protein nanomechanics. **e** A mutation (indicated by an asterisk) can alter mechanical stability and/or folding of the targeted domain. **f** Phosphorylation-induced changes in persistence length result in modulation of protein nanomechanics, as visualized in these two worm-like chain plots in which persistence length increases in the phosphorylated state (+P) as compared to the non-phosphorylated polypeptide (-P), resulting in protein softening. **g** Worm-like chain plot of a reduced (SH) and disulfide-containing (SS) extended polypeptide, showing marked stiffening induced by oxidation as a consequence of shorter contour length. **h** S-glutathionylation of cryptic cysteines in unfolded domains blocks refolding and therefore softens protein domains.

Polypeptide structure defines how proteins respond to mechanical force. As discussed above, random coil structures behave in a purely elastic manner and are quite easily extended under force, while folded structures have intrinsic mechanical resistance. For some proteins with mechanical function, cells can express more than one isoform differing in the proportion of random coil and folded domains (Figure 4a). A paradigmatic example is titin, for which a variety of isoforms can be expressed in myocytes contributing to the passive mechanical properties of muscle tissue (Cazorla et al. 2000; Freiburg et al. 2000; Neagoe et al. 2003). In addition, different protein folds show distinct mechanical properties. Typically, α-helical proteins have low mechanical stability, while β-sheet-containing polypeptides are more resistant to mechanical unfolding, especially if a parallel β-strand mechanical clamp is present (Figure 4b) (Sulkowska and Cieplak 2007). This different behavior of folded domains can be explained by the need to break hydrogen bonds to mechanically unfold the polypeptide, which in the case of parallel β-strands must occur simultaneously (Marszalek et al. 1999).

Since mechanical protein unfolding involves the rupture of interactions present in the mechanical clamp, the mechanical stability of a protein is highly dependent on the pulling geometry (Figure 4c) (Brockwell et al. 2003; Carrion-Vazquez et al. 2003; Shank et al. 2010). Hence, if biophysical measurements are to be extrapolated to the in vivo setting, it is important to consider whether native pulling geometries match the experimental ones (Echarri et al. 2019). Similarly, environmental factors that perturb the free energy of a protein can lead to nanomechanical modulation, including temperature (Botello et al. 2009; Popa et al. 2011), pH (Edwards et al. 2021), crowding (Yuan et al. 2008), surrounding ions (Labeit et al. 2003; Muddassir et al. 2018), and osmolytes (Aioanei et al. 2012; Garcia-Manyes et al. 2009b; Popa et al. 2013b) (Figure 4d). Ligand binding can also modulate protein nanomechanics, as demonstrated for metals and protein partners (Cao and Li 2008; Cao et al. 2008a; Cao et al. 2008b; Kotamarthi et al. 2015; Lof et al. 2019; Milles et al. 2018b; Verdorfer and Gaub 2018) (Figure 4d). The interplay between ligand binding and protein mechanics also enables mechanosensing mechanisms based on force-dependent exposure of binding sites (del Rio et al. 2009; Tapia-Rojo et al. 2020a).

Point mutations targeting both mechanical clamps and other protein regions can result in altered polypeptide mechanical stability (Li et al. 2000a; Sadler et al. 2009) or, more unpredictably, in changes in protein folding (Li et al. 2000a) (Figure 4e). Since mutations in force-bearing proteins cause human disease (Nakamura et al. 2011; Schreiber and Kennedy 2013; Yotti et al. 2019), it has been proposed that alteration of protein nanomechanics can contribute to pathogenesis (Anderson et al. 2013; Ma et al. 2009; Suay-Corredera et al. 2021b). Similarly, protein nanomechanics can be profoundly modulated by post-translational modifications (PTMs). Typical PTMs are reversible biochemical additions to proteins, which can be exploited to modulate protein activity in a fast and highly specific manner (Barber and Rinehart 2018). Specifically, the persistence length of random coil regions can be modulated by phosphorylation (Hidalgo et al. 2009; Kruger et al. 2009; Lanzicher et al. 2020) (Figure 4f), while crosslinking modifications such as isopeptide (Alegre-Cebollada et al. 2010; Echelman et al. 2016) or disulfide bonds (Ainavarapu et al. 2007; Carl et al. 2001) reduce the effective contour length of proteins resulting in marked stiffening (Figure 4g). Disulfides can also influence the mechanical stability of a protein in a context-dependent manner (Giganti et al. 2018; Manteca et al. 2017a; Manteca et al. 2017b) and are positive modulators of protein folding (Eckels et al. 2019; Kosuri et al. 2012). Redox modifications other than disulfide bonds, such as S-glutathionylation, are also potent modulators of protein nanomechanics by inhibiting refolding, which softens targeted domains (Figure 4h) (Alegre-Cebollada et al. 2014). Interestingly, redox modifications that control protein structure can be exploited to modulate protein nanomechanics (Peng et al. 2012). The rich interplay between protein biochemistry and nanomechanics is also exemplified by the mechanical effects of disulfide isomerization, which can be trigged by mechanical force resulting in force-dependent nanomechanical modulation (Alegre-Cebollada et al. 2011b; Giganti et al. 2018). Intriguingly, extended polypeptides age in a time scale of minutes to days losing their ability to fold (Valle-Orero et al. 2017b). Although the mechanisms behind this observation are not completely understood, it is possible that accumulating chemical modifications could contribute to the inability of the protein to refold, similar to the effects of S-glutathionylation described above.

## Key examples of proteins under mechanical force

There is a long list of force-bearing proteins involved in force generation, sensing, and transduction whose nanomechanics can be regulated by the mechanisms described above. In the following sections, I review current knowledge on the biological function and mechanical activity of selected force-bearing proteins. Beyond these specific examples, it is important to stress that arguably all proteins are subject to mechanical force when synthesized by the ribosome (Goldman et al. 2015) and when being translocated through narrow channels, for instance during proteasomal degradation (Alegre-Cebollada et al. 2011a; Aubin-Tam et al. 2011; Maillard et al. 2011).

### Muscle proteins

Striated muscle, including both skeletal and cardiac muscle, has been a prime tissue to investigate the function of proteins under load, thanks to its highly ordered structure based on the regular assembly of sarcomeres (Wang et al. 2021). Indeed, muscle has been a traditional model tissue that has led to important discoveries in biology, such as actomyosin contraction and the sliding filament theory (Szent-Gyorgyi 2004), or the electromechanical coupling controlling contraction (Dulhunty 2006). It is remarkable that macroscopic observations, such as the Frank-Starling law describing how the ventricular function of the heart is adjusted to the filling pressure, can be modeled down to molecular events involving components of the sarcomere (John Solaro 2007).

**Figure 5.**
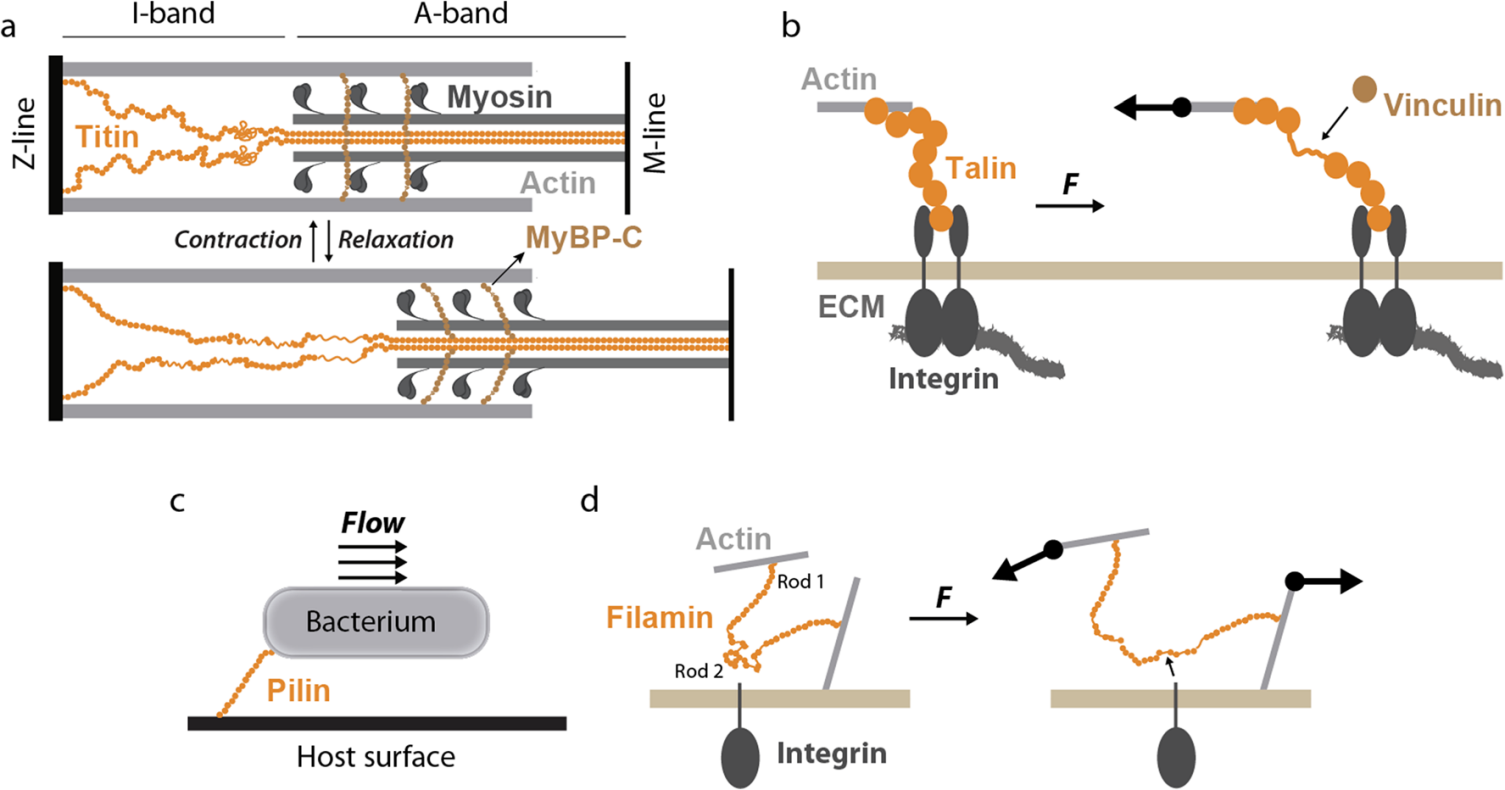
Mechanical functions of key tethered proteins. **a** Representation of one half-sarcomere, highlighting actin-based thin filaments and myosin-containing thick filaments. Titin runs along the sarcomere from the Z- to the M-lines. MyBP-C is located at the A-band of sarcomeres where it interacts both with thin and thick filaments. **b** Talin interacts with actin filaments and adhesion proteins such as integrins. Mechanical force unfolds talin domains resulting in the exposure of binding sites for partners such as vinculin. **c** Adhesive pili in bacteria withstand mechanical perturbations induced by flow and shearing. **d** Filamin dimers bridge actin filaments and therefore are subject to mechanical force when there is relative movement between the filaments. Similar to the talin/vinculin system, mechanical force induces the exposure of cryptic binding sites in rod 2 domains.

Among the different tethered polypeptides in the sarcomere, the giant protein titin (also known as connectin) has captured the attention of muscle researchers since its discovery (Freundt and Linke 2019; LeWinter and Granzier 2010; Maruyama et al. 1977; Wang et al. 1979). It is now well-established that mechanically induced conformational changes in titin’s I-band region are a main determinant of the stiffness of cardiomyocytes, with potential implications in heart physiology and disease (Freundt and Linke 2019; LeWinter and Granzier 2010). Titin localizes to the sarcomeres of striated muscle cells, where it bridges the Z- and M-lines ensuring structural integrity of contracting myocytes (Figure 5a). Beyond this structural function, titin is also a signaling hub through an extensive network of protein interactors (Linke and Hamdani 2014). During the contraction/relaxation cycles of myocytes, titin molecules experience varying mechanical forces that lead to uncoiling/recoiling of the N2-Bus and the PEVK random coil regions and also straightening and unfolding/refolding of serially linked immunoglobulin-like (Ig) domains in the I-band (Figure 5a) (Li et al. 2002; Rivas-Pardo et al. 2020). In addition to their contribution to sarcomere stiffness, it is important to realize that these force-induced conformational changes can also alter the binding affinities for interactors, including signaling and effector molecules such as transcription factors. For instance, there is an inactive kinase domain in the M-line region of titin that is predicted to become activated under mechanical load (Puchner et al. 2008). More recently, the role of folding transitions during active contraction of sarcomeres has been proposed (Rivas-Pardo et al. 2016) and debated (Bianco et al. 2016; Eckels et al. 2018).

The fundamental role of titin for the fitness of myocytes is exemplified by the following facts: (i) mutations in titin are the most common cause of human dilated cardiomyopathy (Herman et al. 2012) and are also responsible for several musculoskeletal conditions (Savarese et al. 2016); (ii) titin deficiency in mice causes embryonic lethality (Gotthardt et al. 2003; Radke et al. 2019); (iii) titin-deficient cells cannot produce functional sarcomeres and do not fully differentiate into myocytes (Hinson et al. 2015; Musa et al. 2006); and (iv) titin is exquisitely regulated, both at the transcriptional and the post-translational levels (LeWinter and Granzier 2010; Linke and Hamdani 2014). At the transcriptional level, titin primary RNA transcripts undergo complex alternative splicing, as introduced in the previous section. There are two major titin isoforms in the adult myocardium, N2BA and N2B. The N2BA isoform is softer than N2B because it is longer and contains a higher proportion of random coil regions. Modulation of the content of titin isoforms is well documented under several physiological and pathological situations, resulting in changes of the mechanical output of the tissue as in the case of hypertensive cardiomyopathy (Linke and Hamdani 2014). A more rapid adjustment of the mechanical properties of titin can be achieved by post-translational modifications like phosphorylation, acetylation, and oxidation, which in turn can also be influenced by force (Abdellatif et al. 2021; Herrero-Galán et al. 2020; Herrero-Galán et al. 2019; Huang et al. 2020; Linke and Hamdani 2014; Loescher et al. 2020).

As for other proteins under mechanical load, most of what we know about the mechanical role of titin in striated muscles derives from correlative observations. To provide mechanistic insights in vivo, a few mouse lines in which specific mechanically active segments of titin are constitutively knocked out have been characterized. The targeted regions include the N2-Bus and the PEVK random coil regions (Brynnel et al. 2018; Granzier et al. 2009; Radke et al. 2007), the proximal tandem Ig domains (Chung et al. 2013), and the I-A band junction (Granzier et al. 2014). Overall, these models have shown that the myocyte is quite adaptable to chronic perturbations of titin mechanical architecture through a range of compensatory changes. For instance, deletion of several PEVK exons in skeletal muscle leads to reduced sarcomere length, which restores the force experienced by titin molecules (Brynnel et al. 2018). At the organismal level, these models show a range of phenotypes, including diastolic dysfunction, atrophy, ventricle dilatation, and hypertrophy, which are hypothesized to result from altered protein-protein interactions and faulty mechanosensing (Granzier et al. 2009). In conclusion, results with available mouse models suggest that dedicated mechanobiochemical mechanisms of the myocyte ensure proper activity of titin, but their molecular basis remains elusive.

Other sarcomeric proteins are also under mechanical load in vivo. For instance, myosin-binding protein C (MyBP-C), located in the C-zone of sarcomeres, interacts with both thick and thin filaments in such a manner that myofilament sliding is predicted to generate load on the protein (Previs et al. 2012) (Figure 5a). Although MyBP-C is much smaller than titin, the mechanical organization in random coil structures and serially linked folded domains is very similar (Karsai et al. 2011), as in other sarcomere components such as the M-line protein myomesin (Berkemeier et al. 2011) and obscurin (Manring et al. 2017).

Mutations in titin and MyBP-C are well-established inducers of different forms of human cardiomyopathy (Harris et al. 2011; Herman et al. 2012). However, the pathomechanisms induced by these mutations remain incompletely understood. Prompted by the sophisticated nanomechanical design of these proteins and the fact that mutations can change protein nanomechanics, there has been an interest in examining if disease-causing variants can alter the nanomechanics of titin and MyBP-C. Specifically, arrhythmogenic-cardiomyopathy-causing mutation T16I was found to decrease the mechanical stability of the Ig10 domain of titin (Anderson et al. 2013), although this effect is concomitant with a marked decrease in thermodynamic stability (Bogomolovas et al. 2016). More recently, nanomechanical alterations in mutant cardiac MyBP-C that cause hypertrophic cardiomyopathy have been detected. These alterations occur in the absence of other pathogenic molecular phenotypes (Suay-Corredera et al. 2021a; Suay-Corredera et al. 2021b). A decrease in mechanical stability at low forces was detected for mutant R495W, which could reduce the braking force exerted by mutant MyBP-C on actomyosin gliding. The same study reported increased mechanical folding of mutant R502Q.

### Adhesion proteins

Protein nanomechanics is key for cell-cell and cell-ECM interaction and adhesion, so it comes as no surprise that proteins belonging to adhesion structures show highly specialized nanomechanical architectures. A paradigmatic example is found in how talin contributes to ECM rigidity sensing by cells. Talin is associated to focal adhesions, where it bridges the intracellular region of integrins and actin filaments (Figure 5b) (Klapholz and Brown 2017). The biphasic mechanosensing response of cells to ECM stiffness can be explained by considering the interplay between force-dependent ECM binding kinetics and the reinforcement of focal adhesions induced by talin unfolding (del Rio et al. 2009; Elosegui-Artola et al. 2016; Haining et al. 2016). In simple terms, when the stiffness of the ECM is high, pulling forces generated by polymerizing actin induce the exposure of cryptic binding sites in talin, which can be recognized by adaptor proteins such as vinculin reinforcing focal adhesions.

In addition to cytoskeleton-associated proteins like talin, cell-ECM adhesion is arguably dependent on the nanomechanics of cell membrane adhesive proteins (Ju et al. 2016; Mikulska-Ruminska et al. 2017; Perez-Jimenez et al. 2014) and of constituent proteins of the ECM, including fibronectin (Oberhauser et al. 2002; Smith et al. 2007) and tenascin (Oberhauser et al. 1998) (Figure 1). Similar to titin, the structure of these ECM proteins is based on serially linked folded domains, suggesting that equivalent modes of protein nanomechanics modulation also target the ECM. Recent experiments suggest that, as for titin kinase, focal adhesion kinase can also be activated by mechanical force (Bauer et al. 2019). Similar mechanosensing systems involving tethered proteins like α-catenin and cadherins ensure cell-cell adhesion (Leckband and de Rooij 2014).

Bacteria also take advantage of refined protein nanomechanics to ensure efficient adhesion to target tissues (to sustain infection) or substrates (for instance to enable catalytic processing of nutrients) (Figure 5c). For instance, micrometer-long pili in Gram positive bacteria are composed by a linear covalent assembly of pilin monomers on the bacterial surface (Mandlik et al. 2008). It is remarkable that the domain organization of titin, ECM proteins, and Gram positive pili is so similar despite their very different biosynthesis. Two distinctive features are found in Gram positive pili. First, they are covalently capped with highly specific adhesive pilins. These capping pilins can contain reactive chemical groups that can be modulated by force to ensure covalent, but reversible, binding to their targets (Alonso-Caballero et al. 2021; Echelman et al. 2017). In addition, structural pilins can contain self-catalyzed, intradomain isopeptide bonds. These bonds can block totally (Alegre-Cebollada et al. 2010) or partially (Echelman et al. 2016) pilin mechanical unfolding. In the latter case, isopeptides enable very fast mechanical refolding, which is compatible with a shock-absorber role for isopeptide-containing pilins.

A common characteristic of adhesins from Gram positive bacteria is their strong resistance to mechanical unfolding, which can also be achieved by mechanical architectures involving non-covalent bonds. For instance, the B domains in *Staphylococcal* adhesins reach covalent-like mechanical stability via coordination of calcium ions (Milles et al. 2018b). In addition, the structure of cohesin domains present in cellulosomal complexes makes them highly resistant to mechanical unfolding (Valbuena et al. 2009; Verdorfer et al. 2017). Altogether, these results suggest strong evolutionary pressure that leads to convergent mechanisms ensuring high mechanical stability of Gram positive adhesins. It is tempting to hypothesize that such mechanically strong folds help bacteria remain attached to their targets during mechanical insults, such as coughing or brushing. However, it remains unclear why Gram negative bacteria show adhesive systems with very different mechanical architectures. For instance, *E. coli* type I pili are built from non-covalent helical assembly of monomers and rely on helix uncoiling and recoiling to accommodate changes in flow (Forero et al. 2006; Miller et al. 2006). The mechanical stability of constituent pilin domains, although lower than in Gram positive counterparts, is high enough to ensure uncoiling of pili (Alonso-Caballero et al. 2018). Most probably, different mechanical architectures of adhesins in Gram positive and negative bacteria reflect adaptation to their specific mechanical niches (Persat et al. 2015).

### Other proteins under mechanical load

Other proteins beyond those participating in muscle contraction or cell adhesion operate under mechanical load. The function of these proteins can involve modulation of cytoskeletal filaments by crosslinking, structural support of organelles, or flow sensing. I have chosen three specific examples (filamin, lamin and von Willebrand factor) to illustrate how protein nanomechanics contribute to these seemingly different mechanical functions, although many other mechanical proteins fall in any of these categories (Johnson et al. 2007; Le et al. 2018; Ramm et al. 2014; Sawada et al. 2006).

Filamins are dimeric proteins able to crosslink actin filaments at different locations in the cell (Razinia et al. 2012). Similar to titin, filamin is composed by serially linked Ig domains, although two different interdomain arrangements are present (Figure 5d). While domains 1–15 (rod 1) show a mostly linear arrangement, domains 16–24 (rod 2) are more compact. X-ray crystallography has uncovered a peculiar arrangement of β-strands in domains belonging to rod 2, by which some Ig folds are completed by β-strands that extend from adjacent domains (Lad et al. 2007). This structural arrangement has been proposed to play mechanosensory roles in filamin A. In particular, the β-strand extending from Ig20 that completes domain Ig21 can be peeled off by low mechanical forces, resulting in the exposure of binding sites for several downstream signaling partners like the cytoplasmic tails of integrins (Figure 5d) (Rognoni et al. 2012). The mechanical stability of the Ig domains of filamin A has been measured using both AFM (Furuike et al. 2001) and MT (Chen et al. 2011).

Lamin proteins are fundamental for the mechanical integrity of the nuclear envelope (Gruenbaum and Foisner 2015) and the genome (Cho et al. 2019). Lamin A self-associates through specific multimerization regions that lead to a meshwork structure (Gruenbaum and Foisner 2015). Interestingly, the levels of lamin A in the nucleus correlate with the stiffness of the ECM (Swift et al. 2013). It remains to be investigated how the different lamin domains contribute to the non-linear mechanics of lamin filaments (Bera et al. 2014, 2016; Sapra et al. 2020).

Von Willebrand factor is a sensor of blood shear and is required for normal hemostasis (Sadler 1998). Molecular mechanisms sustaining shear-flow-sensing involve force-induced conformational changes and exposure of cryptic cleavage sites, which ensure timely mechanical control of the coagulation cascade (Arce et al. 2021; Fu et al. 2017; Lof et al. 2019; Springer 2014; Zhang et al. 2009).

## Moving the protein nanomechanics field from correlations to causality

Newton’s law of universal gravitation ensures that, absent of any other prevailing forces, if I let go of a ball, it will drop and hit the ground. The subsequent effects are more difficult to predict and depend on the specific type of ball, whether the ground is flat or not, or if I am playing in the finals of the basketball World Cup. This example mirrors the situation currently faced when trying to understand the contribution of protein nanomechanics to many aspects of physiology. As elaborated in the previous sections, single-molecule biophysics approaches have shown that proteins under mechanical force experience reversible conformational changes, which can be exploited by biological systems to trigger downstream biochemical signals. However, the study of mechanosensing by force-bearing proteins currently faces challenges derived from the difficulties associated with modulating protein nanomechanics in living cells. For instance, biophysical experiments have demonstrated that mechanical force induces extended states of talin that expose new binding sites (del Rio et al. 2009; Tapia-Rojo et al. 2020a), but it remains impossible to examine how force modulates the interactome of the protein in a cellular context. To overcome current barriers to mechanistic progress, we need to develop tools able to modulate protein nanomechanics in living cells (Guck 2019).

**Figure 6.**
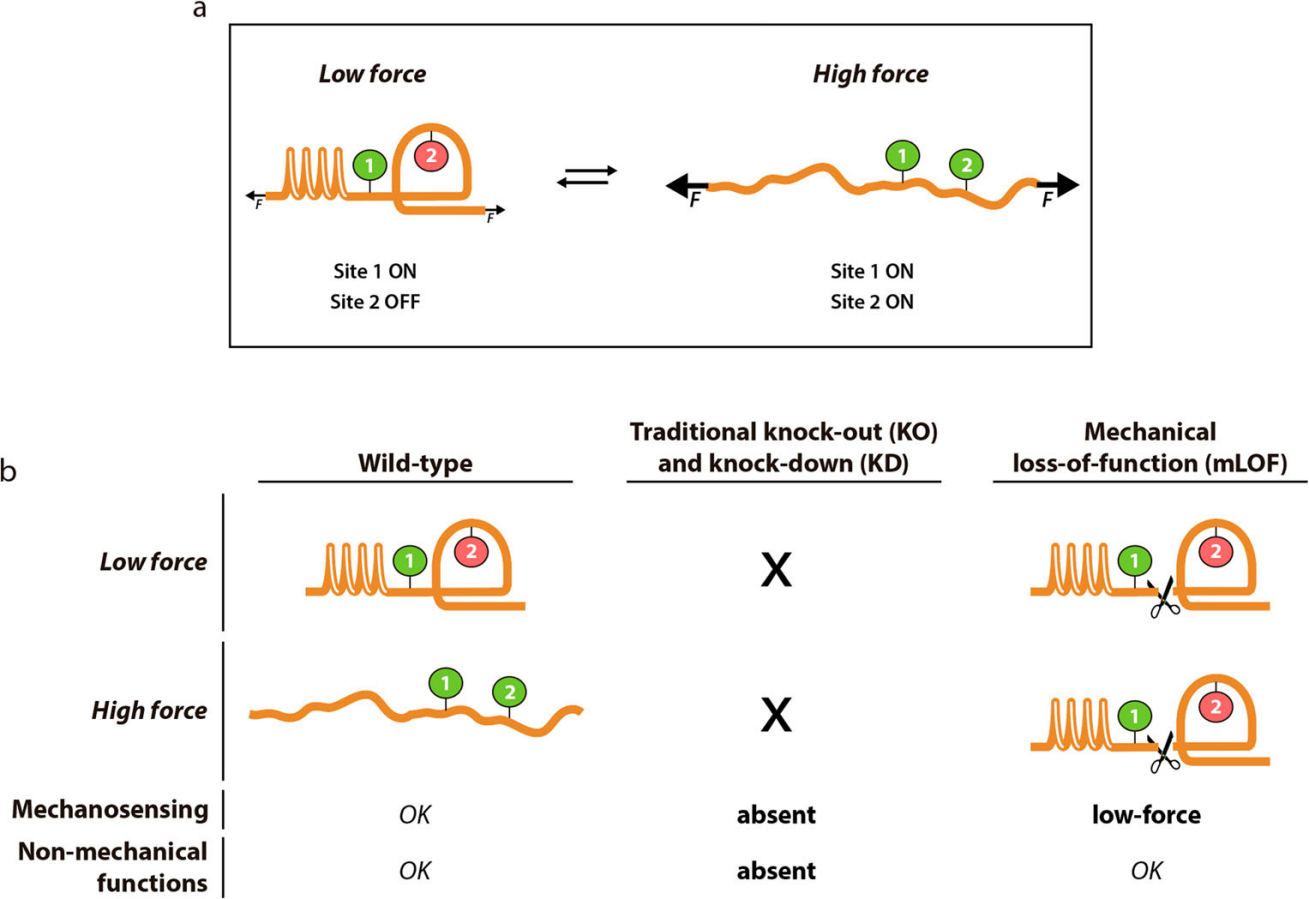
Mechanical loss-of-function (mLOF) to study tethered proteins. **a** A generic force-bearing, mechanosensing protein containing both exposed (1) and cryptic (2) signaling sites. The activity of site 2 is force dependent since mechanical load induces its exposure via domain unfolding. **b** Traditional KO/KD systems get rid of the targeted protein, which interferes both with its mechanical and non-mechanical functions (alterations compared to wild-type are indicated in bold type at the bottom). In contrast, mLOF probes protein mechanical functions specifically by cleaving the protein and locking it in a low-force mechanosensing state.

A number of innovative approaches have been implemented to examine the role of protein nanomechanics in physiology. These strategies are typically multidisciplinary, requiring integration of cell biology, tissue physiology, and biochemical and biophysical experiments through modeling and computer simulations (Elosegui-Artola et al. 2016; Escribano et al. 2018; Fu et al. 2017; Giganti et al. 2018; Li et al. 2002; Rahikainen et al. 2017; Schwarz 2017; Shamsan and Odde 2019). However, these methods face limitations. For instance, Förster resonance energy transfer (FRET) sensors can quantify mechanical tension across molecules, making modeling more accurate, but cannot modulate protein nanomechanics in living cells (Cost et al. 2019; Ham et al. 2019; Lemke et al. 2019; Roca-Cusachs et al. 2017). Mechanistic knock-out (KO), knock-down (KD), and overexpression tools can be used to alter the levels of mechano-active proteins; however, these strategies also interfere with non-mechanical functions, potentially complicating the interpretation of results (Figure 6).

Emerging efforts to put protein nanomechanics in biological context include the acute in vitro expression of mutants with altered mechanical properties. This strategy has uncovered that protein nuclear import depends on the nanomechanics of the cargo, but not on its thermodynamic stability, a finding that can be key for mechano-active transcription factors such as YAP (Elosegui-Artola et al. 2017; Elosegui-Artola et al. 2016; Infante et al. 2019). It has also become possible to control mechanosensitive cell-surface receptors by nanoparticles in vitro (Seo et al. 2016), a method that could potentially be applied also to intracellular proteins. Very recently, strategies based on the cleavage of mechanical proteins using light-sensitive domains (Endo et al. 2019) or specific proteases (Napierski et al. 2020; Rivas-Pardo et al. 2020) have been demonstrated. In these approaches, specific proteolysis ceases force transduction through the targeted protein, which blocks force-induced protein conformational changes. Results so far are encouraging. For instance, severing a photocleavable cadherin in vitro results in attenuated mechanotransduction at intercellular junctions (Endo et al. 2019). TEV-protease-mediated cleavage of cardiac MyBP-C causes mechanical dysregulation of skinned cardiomyocytes leading to spontaneous contractile oscillations (Napierski et al. 2020). In a similar approach, TEV-protease severing of titin led to specific quantification of the passive force generated by the protein in skinned myocytes (Rivas-Pardo et al. 2020), as well as its contribution to sarcomere stability during active contraction (Li et al. 2020).

The results described above demonstrate that these new mechanical loss-of-function (mLOF) tools make it possible to evaluate biological response following acute protein nanomechanical modulation (Figure 6). A number of limitations remain and will need to be addressed to realize the full potential of mLOF tools. For instance, light-induced cleavage can be difficult to implement in vivo due to poor penetration of light in tissues, while protease-based methods require the controlled expression of proteases by living cells. However, the use of different specific proteases, including TEV, to control protein activity in vitro and in vivo is well documented and results in no noticeable off-target effects (Chung and Lin 2020; Harder et al. 2008; Kono et al. 2014). An important limitation is the need to genetically engineer cleavable sequences into the protein of interest, which in the case of large proteins like titin requires laborious editing of endogenous loci. Approaches based on engineering protease specificity (Huang et al. 2016; Pethe et al. 2019) or on drugs that modulate protein nanomechanics specifically are enticing alternatives to be explored.

## Concluding remarks

The field of protein nanomechanics has produced seminal contributions regarding the behavior of proteins under a pulling force, yielding highly accurate models of how mechanical force shapes the free energy of proteins. This remarkable success has been enabled by key experimental and theoretical developments over the last 25 years. Nowadays, nanomechanical probing of single proteins has become routine in many laboratories. Current biophysical experiments often investigate how protein nanomechanics is modulated by factors present in living tissues or by mutations, including those that cause human disease. However, instead of replicating in vivo conditions in single-molecule force spectroscopy experiments, there is a growing interest to do just the opposite, i.e., developing strategies to modulate protein nanomechanics in living systems to examine biological response. Among them, I anticipate that protein-cleavage mLOF tools will open new windows of opportunity to investigate the connection between protein nanomechanics and other biological functions. Efforts in this direction include the generation of cell and animal models in which protein nanomechanics can be specifically modulated at will.

## Biography: Jorge Alegre-Cebollada

Prof. Alegre-Cebollada leads the “Molecular Mechanics of the Cardiovascular System” group at CNIC. His laboratory is devoted to understanding how mechanical forces determine muscle function at the molecular, cellular, tissue, and organismal levels. Jorge was initially trained as a protein biochemist, studying how sea anemone actinoporins interact with lipid membranes to exert their cytotoxic action. His PhD thesis work, defended in 2008, was supervised by Prof. Álvaro Martínez-del-Pozo and Prof. José G. Gavilanes from the Department of Biochemistry and Molecular Biology at Complutense University of Madrid, Spain. During this time, Jorge learned about molecular biology, protein biochemistry, and lipid-protein interactions in a very nurturing environment, which he now tries to emulate in his own laboratory.

After completing his PhD, Jorge was lucky to be accepted at the laboratory of Prof. Julio M. Fernández at Columbia University in New York. Fascinated by the extraordinary resolution of single-molecule AFM and the quantitative treatment of AFM data, he remembers reading with awe the 2006 PNAS paper in which Prof. Fernández and colleagues described the force-dependent kinetics of disulfide cleavage using force-clamp AFM (Wiita et al. 2006). During his period at Columbia (2008-2014), Jorge led several projects at the crossroads between protein biochemistry and nanomechanics, including the demonstration that some pilin proteins are mechanically inextensible due to the presence of self-catalyzed isopeptide bonds, the first direct observation of disulfide isomerization reactions in real time and the realization that post-translational modification of cryptic residues is a potent modulator of protein nanomechanics.

The Fernandez lab was highly interdisciplinary and attracted very talented scientists. There was plenty of discussion about ongoing and future projects. Crazy ideas were always welcome, especially if they were conceived while playing foosball or ping-pong. One of them was brewed by Julio and Jorge while having coffee at the downstairs cafe. The idea was to produce a genetically engineered mouse model to be able to probe native titin nanomechanics using HaloTag technology and TEV protease. Julio and Jorge had no previous experience with in vivo work, but Julio decided to give it a try. A few years later, and thanks to the invaluable input from several colleagues, they published what arguably is among the first mLOF models (Rivas-Pardo et al. 2020). Aware from his experience at Columbia that groundbreaking ideas often appear in unconventional settings, Jorge now encourages unusual forms of discussion in Madrid, to the occasional dismay of some regulators.

Starting in 2014, Jorge’s independent research has focused on achieving an understanding of how protein nanomechanics is integrated within biological systems. His laboratory still exploits single-molecule methods, but thanks to the outstanding facilities at CNIC the team has added cell and in vivo methods to their portfolio of experimental approaches. Current active research projects in Jorge’s laboratory include pathomechanisms of mutations in mechanical proteins that cause cardiomyopathy, functional relevance of post-translational modifications that modulate protein nanomechanics, engineering of protein hydrogels to probe specific contributions of ECM mechanics to cell biology, and the development of in vivo mLOF tools.

Jorge is very proud to be the recipient of the Michèle Auger Award for Young Scientists’ Independent Research 2021. Other honors include the election as a member of the Spanish Young Academy in 2021, the nomination as Arsenic in the Periodic Table of Younger Chemists by the IUPAC in 2018, and the award to the best young biophysicist by the Spanish Biophysical Society in 2013.

Jorge is a proud father of three young girls, Paola (8), Irene (5), and Nadia (8 months old). It follows that life is quite busy for him and his wife Lidia, a computational chemist devoted to innovation consulting. However, thanks to Lidia’s invaluable support, Jorge always finds the time for his weekly yoga practice, in which he considers himself an eternal beginner (although already able to do head stands!). Jorge thinks that yoga practice may be the best way of inducing softening redox modifications of titin in vivo, although he is still thinking about the best experiment to prove it. He considers that convincing Julio Fernández to practice yoga is one of his most remarkable professional achievements. Jorge is very fond of nature and always moans about not spending more time in the outdoors.

## The Michèle Auger Award for Young Scientists’ Independent Research

In late 2018, long time Editorial Board Member of Biophysical Reviews journal, Professor Michèle Auger, sadly succumbed to illness. As a mark of our respect for Michèle, the Biophysical Reviews’ Editorial Board, together with the kind support of Springer-Nature Corporation, created a perpetual memorial award in honor of her life and service. The “Michèle Auger Award for Young Scientists’ Independent Research” is to be granted each year to a single candidate performing biophysical research, who at the time of application is under 40 years of age. The award consists of a plaque and a free personal subscription to the journal along with an invitation to submit a single author review article to Biophysical Reviews.

## Biography: Michèle Auger (1963–2018)

Born in GrandMère, Quebec, and raised in TroisRivières, Michèle Auger enrolled first in biophysics at the Université du Québec à Trois-Rivières, only to later transfer to chemistry. After obtaining her B. Sc. in 1985, Michèle joined the group of Prof. Ian C.P. Smith at the University of Ottawa/National Research Council of Canada to pursue her Ph.D. studies in biophysics. After graduating from the University of Ottawa in 1990, Michèle refined her skills in solid state NMR as a postdoctoral fellow in the group of Prof. Robert G. Griffin at the Massachusetts Institute of Technology. Michèle joined the Department of Chemistry at the University of Laval in 1991 as an assistant professor and recipient of an NSERC Women’s Faculty Award. She was promoted to associate professor in 1996 and then professor in 2000 where she remained until 2018. Michèle’s research involved using solid state NMR to study (i) the interaction of proteins, peptides, and drugs with phospholipid membranes and (ii) biopolymers such as spider silk. Michèle served internationally on the Council of the International Union of Pure and Applied Biophysics from 2011 to 2017 and was an editorial board member of Biophysical Reviews journal from 2011 to 2018. Brilliant, creative, and dedicated to the scientific and academic communities, Michèle displayed admirable professional, ethical, and leadership qualities. She is remembered as a dedicated, generous, and inspirational scientist who touched the lives of many through her friendship, teaching, and kindness.

(This short foreword is adapted from a longer memorial published on the IUPAB Newsletter #70: http://iupab.org/wp-content/uploads/2019/02/IUPAB-news-70-2-1.pdf)
